# Human beta defensins 3 and 6 serve as diagnostic tools for identifying sepsis and pneumonia

**DOI:** 10.3389/fimmu.2026.1854226

**Published:** 2026-08-13

**Authors:** Nourah Almansour, Fatema Al-Rashed, Shihab Kochumon, Ali Al Mahmeed, Eman Farid, Khalid Bindayna, Hasan Mohamed Saeed, Rasheed Ahmad

**Affiliations:** 1 Immunology and Microbiology Department, Dasman Diabetes Institute, Kuwait City, Kuwait; 2 Department of Microbiology, Immunology and Infectious Diseases, College of Medicine and Medical Sciences, Arabian Gulf University, Manama, Bahrain; 3 Adult Intensive Care Unit, Salmaniya Hospital, Ministry of Health, Manama, Bahrain

**Keywords:** biomarkers, diagnosis, HBD, pneumonia, sepsis

## Abstract

**Background:**

Sepsis poses significant challenges in early detection and treatment due to its heterogeneous nature and overlaps with other conditions, such as pneumonia. Identifying reliable biomarkers is crucial for intervention and outcomes.

**Methods:**

A total of 190 adults, aged 18 years and above, categorized into four groups: sepsis (n=50), pneumonia (n=53), pneumonia with sepsis (n=34), and healthy controls (n=53). Patients received treatments in the ICU, and the healthy were donors from blood bank. ELISA and qRT-PCR utilized to assess protein and mRNA levels in peripheral blood samples from all participants. ROC curve analyses conducted to determine diagnostic accuracy.

**Results:**

mRNA and protein levels of HBD-3 and -6 significantly elevated in all patients compared to healthy. ROC analysis revealed high sensitivity but low to moderate specificity for HBD-3 and -6. HBD-6 emerged as a promising biomarker for sepsis patients with or without pneumonia.

**Conclusion:**

HBD-3 mRNA and HBD-6 protein could be potential as diagnostic markers for sepsis and pneumonia. Despite limitations such as sample size and technical constraints, this study emphasizes the potential of using HBDs as biomarkers, requiring further research to enhance their reliability and clinical usefulness.

## Introduction

Sepsis is a complex and potentially fatal syndrome that results from a dysregulated inflammatory response to infection. According to the Society of Critical Care Medicine (SCCM), the most recent classification, Sepsis-3, defines sepsis as life-threatening organ dysfunction caused by a dysregulated host response to infection. While the immune system’s initial goal is to eliminate invading pathogens, excessive immune activation can cause widespread tissue damage, lead to multiple organ dysfunction syndrome (MODS), and ultimately result in death (1). Sepsis has long challenged healthcare systems due to its complex pathophysiology and persistently high rates of morbidity and mortality. According to the World Health Organization (WHO), sepsis is responsible for up to 20% of global deaths, ranking it among the leading causes of mortality worldwide (2). Its impact is particularly pronounced in Gulf Cooperation Council (GCC) countries, including the Kingdom of Saudi Arabia and the State of Kuwait. A 2020 statistical analysis reported that sepsis was responsible for 41.7% of deaths among 12 cases of transplant-related strongyloidiasis, underscoring its severe consequences in vulnerable patient populations (3). Early identification of sepsis is crucial for reducing morbidity and mortality. Upon admission to the intensive care unit, healthcare providers commonly use the Sequential Organ Failure Assessment (SOFA) score—formerly known as the sepsis-related organ failure assessment, to aid in the diagnosis of sepsis. This scoring system evaluates dysfunction across multiple organ systems based on clinical observations, laboratory results, and therapeutic interventions. However, the parameters assessed by SOFA are not specific to sepsis, which can complicate timely and accurate diagnosis (1). Currently, there is no definitive diagnostic test for sepsis; therefore, clinicians rely on a combination of laboratory tests and clinical indicators to make a timely diagnosis (4). The diagnosis of sepsis involves a range of essential biomarkers that are critical for clinical identification. Among the most commonly used are procalcitonin (PCT) and C-reactive protein (CRP), which are routinely employed in clinical laboratory settings to evaluate infection and inflammation (5, 6). Although well-established, these biomarkers lack the specificity and sensitivity needed for optimal diagnostic accuracy (7). Monocyte Distribution Width (MDW) has emerged as a potential diagnostic marker for sepsis; however, it is not exclusively specific to the condition (8, 9). ​ Similarly, Pancreatic Stone Protein (PSP) has shown promise as a valuable biomarker for sepsis diagnosis, but elevated PSP levels can also be observed in other inflammatory conditions. This lack of exclusivity highlights the importance of interpreting PSP in conjunction with other clinical findings and diagnostic tests to ensure an accurate diagnosis of sepsis (10). Therefore, there remains a need for more accurate biomarkers for the early diagnosis of sepsis.

In recent years, growing attention has been directed toward human beta-defensins (HBDs) as potential diagnostic markers for sepsis and related conditions. HBDs are a vital component of the innate immune system, playing a central role in the body’s defense against microbial invasion. HBDs exhibit broad-spectrum antimicrobial activity against bacteria, fungi, and viruses and play an important role in innate immune defense (11). The primary objective of this research is to investigate and evaluate the diagnostic significance of human beta-defensins 3 and 6 (HBD-3 and HBD-6) in the context of sepsis, pneumonia, and pneumonia-associated sepsis.

Therefore, this study aimed to investigate the diagnostic significance of HBD-3 and HBD-6 in patients with sepsis, pneumonia, and pneumonia-associated sepsis by evaluating their mRNA and protein expression and comparing their diagnostic performance with established biomarkers.

## Materials and methods

### Study design

This study was conducted at Salmaniya Medical Complex in the Kingdom of Bahrain between February 2018 and September 2019. A total of 190 adult participants, aged 18 years and above, were enrolled and categorized into four distinct groups. These included patients with sepsis syndrome (n = 50), patients with pneumonia (n = 53), and patients with pneumonia-associated sepsis (n = 34), all of whom were admitted to the intensive care unit (ICU) for treatment. The control group consisted of healthy individuals (n = 53) recruited from the Blood Bank at Salmaniya Medical Complex, with no history of infections or inflammatory diseases. Sample size calculations determined a minimum of 17 participants per group to achieve a statistical significance level of 5% and a power of 80%.

### Participant inclusion/exclusion criteria

Clinical identification of sepsis was based on the presence of infection combined with evidence of organ dysfunction, as indicated by an acute change in the total Sequential Organ Failure Assessment (SOFA) score. The diagnosis followed the criteria established by the American College of Chest Physicians/Society of Critical Care Medicine Consensus Conference Committee (ACCP/SCCM). For patients without prior infection or pre-existing organ dysfunction, a baseline SOFA score of zero was assigned. A SOFA score of two or higher corresponds to an estimated 10% mortality risk in a general hospital population with suspected infection (1). The diagnosis of pneumonia is made by assessing the patient’s symptoms and signs of respiratory tract infection, which may include fever, sputum production, breathlessness, wheezing, chest discomfort or pain, and focal chest signs, along with the severity of the illness and other relevant clinical characteristics. A chest X-ray is also performed to confirm the diagnosis (12, 13). Patient data were obtained from their clinical and laboratory files at the ICU and laboratory of Salmaniya Medical Complex in the Kingdom of Bahrain, with a focus on microbiological results identifying the microorganisms involved. All participants were fully informed about the nature and objectives of the study. Written informed consent was obtained from each participant in accordance with the ethical principles outlined in the Declaration of Helsinki. The study was approved by the Research and Ethics Committee of the College of Medicine and Medical Sciences, Arabian Gulf University, Kingdom of Bahrain (Approval Number: E021-P1-10/17), as well as by the Ministry of Health in the Kingdom of Bahrain (Approval Number: AURS/45/2019).

### Blood sampling

Peripheral whole blood samples were collected from each participant via venipuncture, with two samples obtained from both cases and controls. One sample was drawn into a Tempus™ Blood RNA Tube by Applied Biosystems (3 mL of blood), while the other was collected in an IMPROVACUTER^®^ ethylenediaminetetraacetic acid (EDTA) tube (2 mL of blood). The Tempus™ Blood RNA Tube contains 6 mL of Stabilizing Reagent, which, when mixed with 3 mL of whole blood, facilitates cell lysis, deactivates cellular RNases, and promotes targeted RNA precipitation. This reagent allows for sample preservation for up to five days at room temperature, at least seven days at 4°C, or for extended periods when stored at −80°C (14, 15). Following blood collection, plasma was separated from the EDTA tube by centrifugation at 1000× g for 15 minutes, within a temperature range of 2°C to 8°C. This procedure was completed within 30 minutes of blood collection. The plasma was then divided into aliquots and stored at −80°C until needed for analysis (16).

### RNA extraction

RNA extraction was performed using the Tempus™ Blood RNA Tube by Applied Biosystems and the Stabilized Blood-to-CT™ Nucleic Acid Preparation Kit. The purity of the RNA was assessed by measuring the optical density (OD) 260/280 and 260/230 ratios using the NanoDrop^®^ ND-1000 UV-V spectrophotometer (Thermo Fisher Scientific, Waltham, MA, USA). Additionally, the integrity of the RNA was evaluated through agarose gel electrophoresis.

### cDNA preparation

Total RNA (2 µg) was reverse transcribed into complementary DNA (cDNA) using the Applied Biosystems™ High-Capacity cDNA Reverse Transcription Kit (Catalog number 4368814), following the manufacturer’s instructions. Briefly, 10 µL of RNA was mixed with 10 µL of 2X RT Master Mix, which contained 2 µL of RT Buffer, 0.8 µL of deoxyribonucleotide triphosphate (dNTP) Mix, 2 µL of RT random primers, 1 µL of MultiScribe™ reverse transcriptase, and 4.2 µL of nuclease-free water, yielding a final volume of 20 µL. The reverse transcription (RT) reaction was carried out at 25°C for 10 minutes, followed by incubation at 37°C for 2 hours, and a final incubation at 85°C for 5 minutes. After completion, all cDNA samples were stored at −20°C for subsequent analysis.

### Reverse transcription quantitative polymerase chain reaction

The expression levels of the target genes (HBD-3, HBD-6, PCT, and CRP) were measured by comparing them to the expression of a reference gene, human beta-actin (hB-actin), in both patient blood samples and healthy controls using the 7500 Real-Time PCR System (Applied Biosystems) and reverse transcription quantitative polymerase chain reaction (RT-qPCR). All primers for the target and reference genes were designed based on sequence information from GenBank using NCBI primer design, as shown in **Table 1**. A total reaction volume of 10 µL was prepared by combining 30 ng of cDNA, Master Plus SYBR Green 1 probe (Roche Molecular Biochemical, Germany), forward and reverse primers (50 ng/µL), and nuclease-free water. All experiments were performed in duplicate to ensure consistency in experimental conditions. The cycling conditions included an initial denaturation at 95°C for 10 minutes, followed by 40 cycles of 95°C for 15 seconds and 60°C for 60 seconds. A no-template control was included in every PCR run. The results were analyzed using Sequence Detection Software (SDS), version 1.4 (Applied Biosystems), to calculate the threshold cycle (Ct) values for the target genes. The expression level of each target gene was normalized relative to the expression level of the reference gene, human beta-actin (hB-actin). Data were expressed as fold changes, calculated using the 2−ΔΔCt method (17).

**Table 1:** Oligonucleotide primers used in RT-qPCR (18–27).

Gene	Forward primer sequence (5` - 3`)	Reverse primer sequence (5` - 3`)	Length	Tm (°C)	Accession number
HBD-3	TTGCTCTTCCTGTTTTTGGTG	CGCCTCTGACTCTGCAATAA	85	64	NM_001081551.3
HBD-6	TGCTCTTCTTTCTGACCCCA	CCGACAGCATTTCAGAGACTTC	137	65	NM_152251.4
PCT	ATGGGCTTCCAAAAGTTCTCC	CTTCGTCCTCACTGAGCGTG	139	65	NM_001033953.3
CRP	TGTATCCCTCAAAGCACCGTT	GGACAGTTCCGTGTAGAAGTGG	76	65	NM_001382703.1
HB-Actin	TTGTTACAGGAAGTCCCTTGCC	ATGCTATCACCTCCCCTGTGTG	370	57	NM_001101

### Enzyme-linked immunosorbent assay

The protein concentrations of HBD-3, HBD-6, PCT, and CRP were quantified in all participants using the Sandwich Enzyme-Linked Immunosorbent Assay (ELISA). Commercial ELISA kits for HBD-3 (Catalog No. MBS700471), HBD-6/DEFB106A (Catalog No. MBS9317542), PCT (Catalog No. MBS2882041), and CRP (Catalog No. MBS169223) were purchased from MyBioSource (CA, USA) and used according to the manufacturer’s instructions. Absorbance readings were obtained using the Thermo Electron Corporation Multiskan Spectrum.

### Statistical analysis

Data analysis was conducted using SPSS software, version 23 (IBM Corp., Armonk, NY, USA). The independent samples t-test was used for continuous variables, while the Chi-square (χ²) test was applied to categorical variables to compare differences in demographic, clinical, and gene expression data between patients and healthy controls. Receiver operating characteristic (ROC) curve analysis was performed for each gene to assess diagnostic performance, and areas under the curve (AUCs) were calculated. The AUC quantifies the test’s ability to discriminate between compared groups, with values ranging from 0.5 (non-informative) to 1 (perfect biomarker). Data are presented as mean ± standard error (SE). A p-value of ≤ 0.05 was considered statistically significant.

## Results

### Characteristics of study participants

A total of 190 participants were enrolled in this study, consisting of 53 healthy controls, 50 patients diagnosed with sepsis, 53 with pneumonia, and 34 with both sepsis and pneumonia. The gender distribution varied across the groups: the healthy control group included 51 (96.2%) males and 2 (3.8%) females, while the sepsis group comprised 24 (48%) males and 26 (52%) females. In the pneumonia group, there were 27 (50.9%) males and 26 (49.1%) females. The group with both sepsis and pneumonia exhibited an equal gender distribution, with 17 (50%) males and 17 (50%) females. The age distribution for each group was calculated as mean ± standard error (SE), with values of 39.3 ± 1.3 years for healthy individuals, 53.2 ± 2.3 years for sepsis patients, 58.6 ± 2.9 years for pneumonia patients, and 59.5 ± 3 years for those diagnosed with both sepsis and pneumonia. The age differences among the studied groups were statistically significant (p < 0.0001) when compared to the healthy control group. These demographic characteristics are summarized in **Table 2**.

**Table 2:** Age characteristics of study participants.

		Healthy	Sepsis	Pneumonia	Pneumonia with sepsis
Age	Mean ± SE	39.3 ± 1.3	53.2 ± 2.3	58.6 ± 2.9	59.5 ± 3
	Range (Years)	21 - 55	21 - 75	18 - 89	26 - 86
*P* value			<0.0001	<0.0001	<0.0001
			***	***	***

***P < 0.0001.

**Figure 1:**
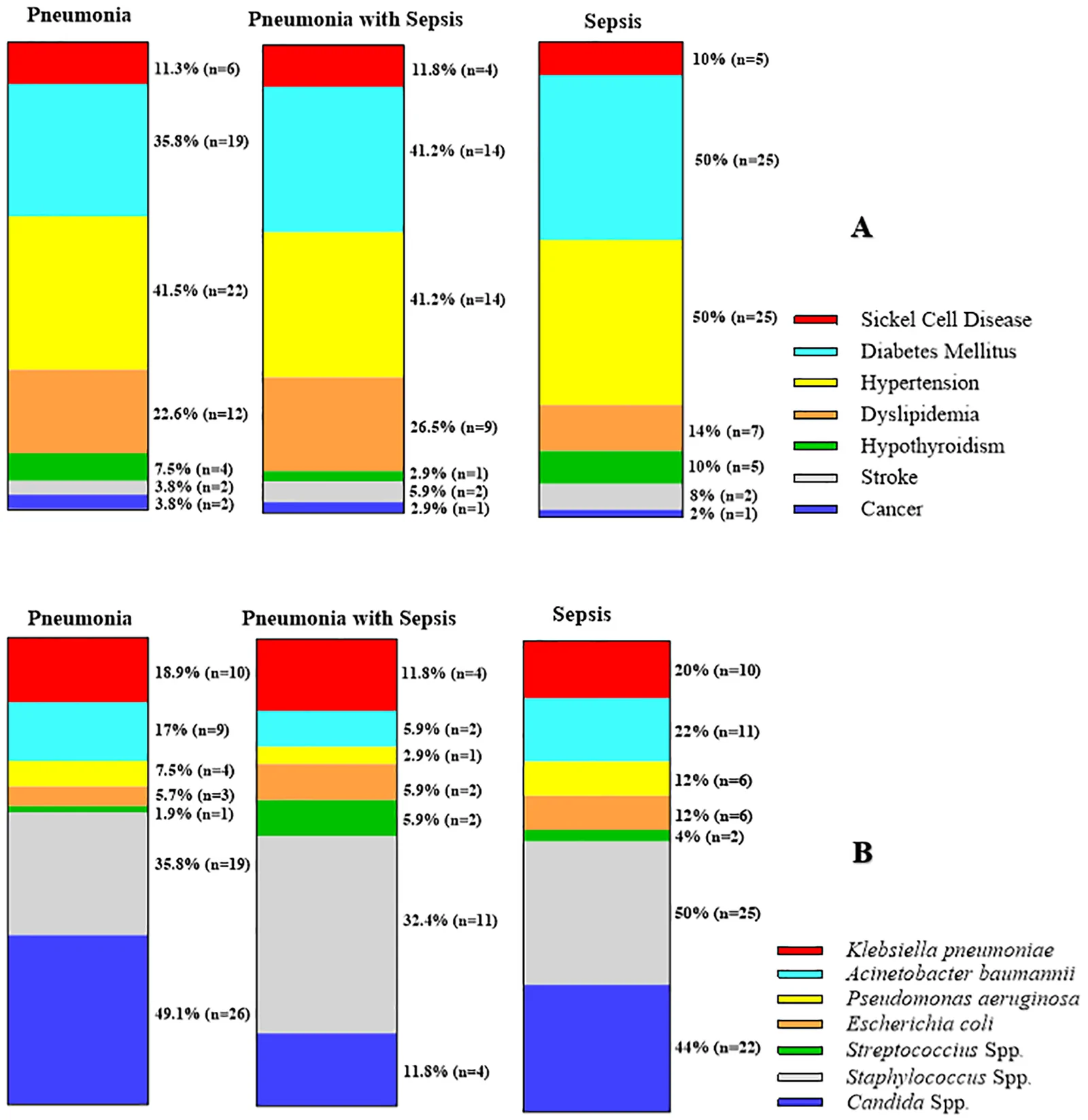
Prevalence of comorbidities and microbiological distribution in sepsis, pneumonia, and co-occurring cases. A total of 190 participants were enrolled in this study which included 53 healthy individuals as controls, 50 diagnosed with sepsis, 53 with pneumonia, and 34 diagnosed with both sepsis and pneumonia. A comprehensive analysis of patient **(A)** comorbidities is shown in (%). **(B)** distribution of infectious microorganisms in patients presented in (%).

Diabetes mellitus and hypertension were the most prevalent comorbidities across all patient groups, followed by dyslipidemia. Other comorbidities, including sickle cell disease and hypothyroidism, were less common. The distribution of comorbidities is summarized in **Figure 1A**. The microbiological profile differed among the patient groups. *Staphylococcus* spp. and *Candida* spp. were the predominant microorganisms, with *Acinetobacter baumannii* and *Klebsiella pneumoniae* also frequently detected (**Figure 1B**).

### HBD gene expression in sepsis, pneumonia, and pneumonia with sepsis

**Figure 2:**
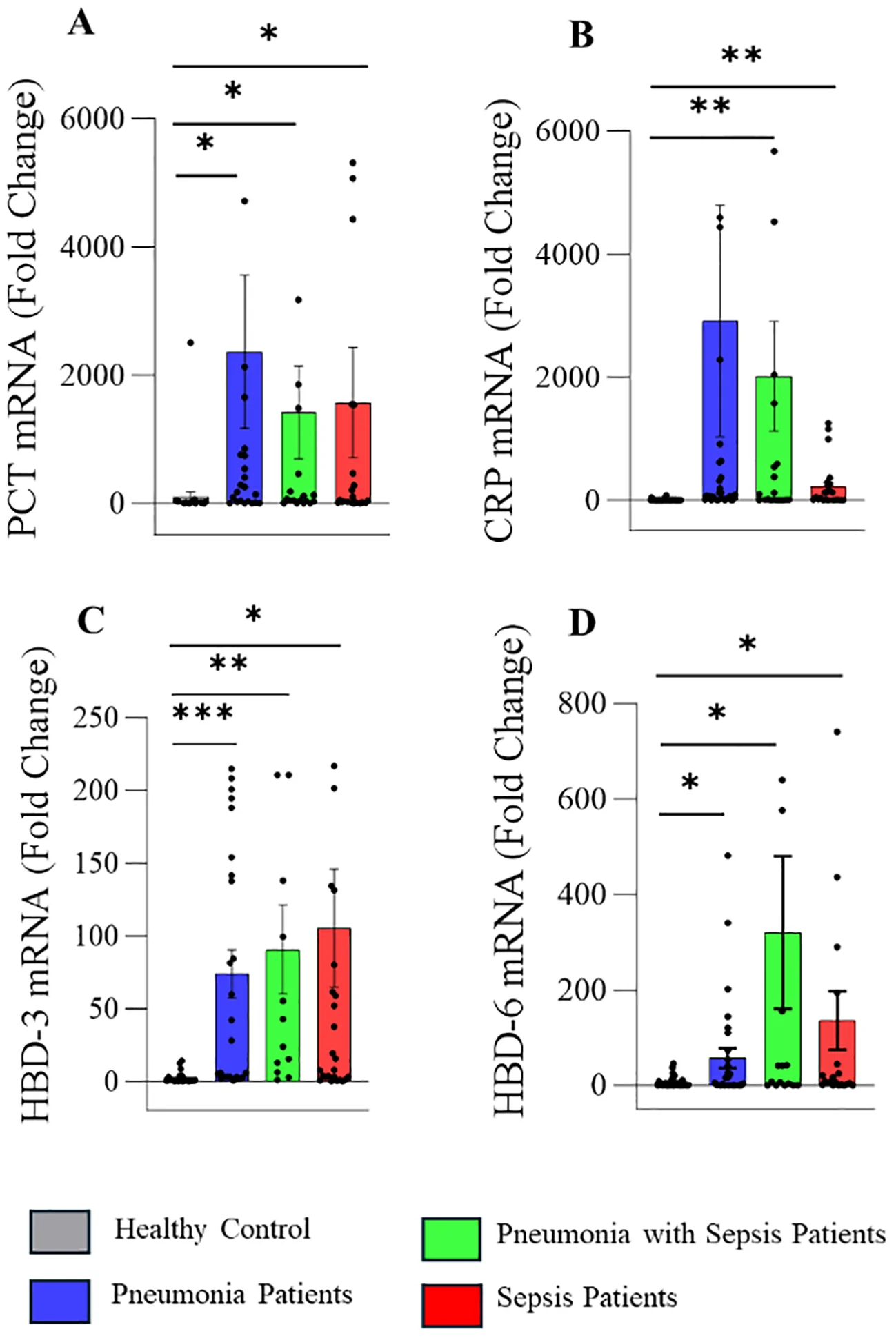
HBD gene expression patterns in sepsis, pneumonia, and co-occurring infections. Peripheral Blood Mononuclear Cells were isolated from each participant. Gene expression analysis was conducted via reverse transcription quantitative polymerase chain reaction (RT-qPCR) to investigate the **(A)** Relative expressions of procalcitonin, PCT, **(B)** Relative expressions of C-reactive protein, CRP, **(C)** Relative expressions of human beta-defensin-3, HBD-3, and **(D)** Relative expressions of human beta-defensin -6, HBD-6, mRNA in patients with sepsis, pneumonia, and pneumonia with sepsis, compared to healthy controls. The expressions of all studied mRNA were quantified relative to human beta-actin (hB-actin). Data are presented as ± SE. *P* ≤ 0.05 was considered statistically significant (*P ≤ 0.05), (**P ≤ 0.001), (***P ≤ 0.0001).

RT-qPCR analysis demonstrated significantly increased HBD-3, HBD-6, PCT, and CRP mRNA expression in all patient groups compared with healthy controls (**Table 3**, **Figure 2**). Pneumonia patients showed the highest PCT and CRP expression, whereas HBD-3 and HBD-6 were elevated across all disease groups.

**Table 3:** Relative quantification (fold change) of mRNA expression of HBD-3, HBD-6, PCT, and CRP in patients and healthy controls.

	Healthy	Sepsis		Pneumonia		Pneumonia with sepsis	
mRNA	Mean ± SE	Mean ± SE	*p* value	Mean ± SE	*p* value	Mean ± SE	*p* value
HBD-3	2.7 ± 0.93	105.4 ± 40.5	0.035 *	73.9 ± 16.8	0.0004 ***	90.8 ± 30.7	0.001 **
HBD-6	6.5 ± 2.14	135.8 ± 61.5	0.017 *	57.1 ± 20.8	0.019 *	320.2 ± 160.2	0.011 *
PCT	7.9 ± 2.28	1569 ± 862.9	0.05 *	2358 ± 1197	0.032 *	1416 ± 722.1	0.017 *
CRP	6.5 ± 2.39	217.1 ± 75.7	0.0014 **	2914 ± 1881	0.111	2013 ± 888.5	0.005 **

*P ≤ 0.05, **P ≤ 0.001.

### HBD’s protein concentrations in the peripheral blood under sepsis, pneumonia, and pneumonia with sepsis

**Figure 3:**
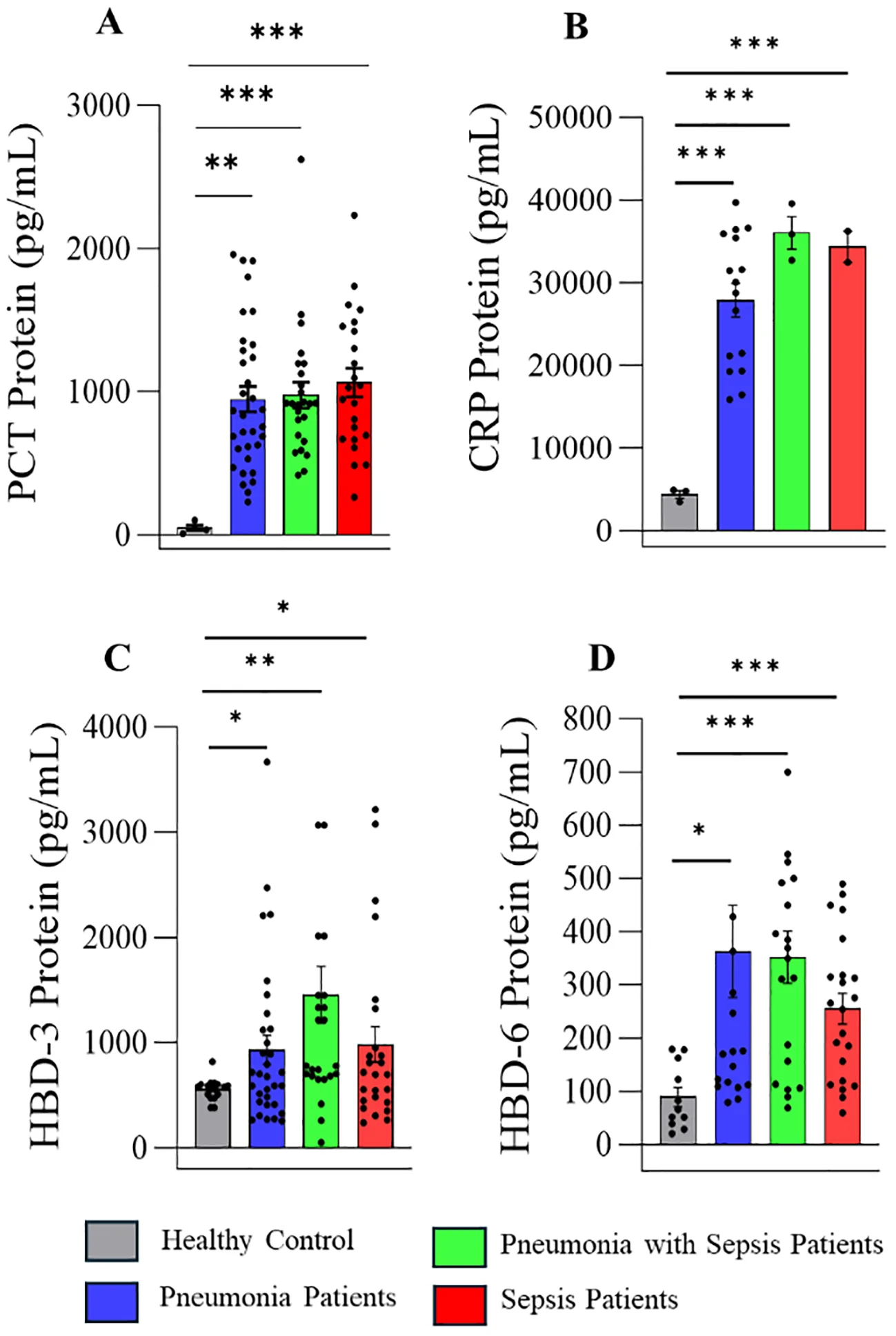
HBD protein secretion patterns in sepsis, pneumonia, and co-occurring Infections. Diagnostic markers were determined in the plasma samples of all participants through use of ELISA **(A)** procalcitonin, PCT (pg/mL), **(B)** C-reactive protein, CRP, (pg/mL) **(C)** human beta-defensin-3, HBD-3(pg/mL), and **(D)** human beta-defensin -6, HBD-6 (pg/mL). Protein concentrations (pg/mL). Data are presented as ± SE. *P* ≤ 0.05 was considered statistically significant (*P ≤ 0.05), (**P ≤ 0.001), (***P ≤ 0.0001).

ELISA confirmed significantly increased HBD-3, HBD-6, PCT, and CRP protein levels in patients compared with healthy controls (**Table 4**, **Figure 3**).

**Table 4:** Protein concentrations of HBD-3, HBD-6, PCT, and CRP (pg/mL) in plasma of patients and healthy controls.

	Healthy	Sepsis		Pneumonia		Pneumonia with sepsis	
Protein	Mean ± SE	Mean ± SE	*p* value	Mean ± SE	*p* value	Mean ± SE	*p* value
HBD-3	554.8 ± 24.38	982 ± 168.4	0.045 *	931.5 ± 136.3	0.05 *	1454 ± 269.5	0.0093 **
HBD-6	89.1 ± 18.25	255.3 ± 28.51	0.0005 ***	362.7 ± 87.3	0.026 *	351.9 ± 49.1	0.0006 ***
PCT	47.9 ± 20.15	1064 ± 100.2	0.0003 ***	945.7 ± 87.9	0.0013 **	974.6 ± 89.9	0.0004 ***
CRP	4369 ± 462.1	34365 ± 1878	0.0003 ***	27869 ± 1998	0.0001 ***	36041 ± 1995	0.0001 ***

*P ≤ 0.05, **P ≤ 0.001, ***P ≤ 0.0001.

### Receiver operating characteristic analysis at gene level

(**Table 5**). ROC analysis demonstrated significant discriminatory power (AUC > 0.7) for the established markers, PCT and CRP, in sepsis patients compared to healthy controls. Both markers exhibited high sensitivity (97%). However, their relatively low specificities (38% for PCT and 46% for CRP). Interestingly, HBD-3 emerged as a promising candidate biomarker, especially for sepsis (AUC = 0.81; 95% CI: 0.69–0.93; *P* = 0.0004), with good sensitivity (95%) and lower specificity (52%). While HBD-3 performed well in pneumonia (AUC = 0.88; 95% CI: 0.78–0.98; *P* < 0.0001) and co-infection (AUC = 0.93; 95% CI: 0.85–0.99; *P* < 0.0001), HBD-6 showed lower discriminatory power across all conditions.

**Table 5:** AUCs of HBD-3, HBD-6, PCT, and CRP mRNAs for patients with sepsis, pneumonia, and pneumonia with sepsis, and healthy controls.

(A) mRNA (Sepsis)	AUC	95% CI		P Values	Sensitivity (%)	Specificity (%)
		Lower Bound	Upper Bound			
HBD-3	0.81	0.69	0.93	0.0004	95	52
HBD-6	0.67	0.5	0.83	0.047	97	24
PCT	0.81	0.70	0.92	< 0.0001	97	38
CRP	0.83	0.72	0.94	< 0.0001	97	46

(B) mRNA (Pneumonia)	AUC	95% CI		*P* Values	Sensitivity (%)	Specificity (%)
		Lower Bound	Upper Bound			
HBD-3	0.88	0.78	0.98	< 0.0001	95	54
HBD-6	0.56	0.44	0.74	0.24	97	28
PCT	0.87	0.78	0.97	< 0.0001	97	67
CRP	0.84	0.74	0.94	< 0.0001	97	44

(C) mRNA (Pneumonia with Sepsis)	AUC	95% CI		*P* Values	Sensitivity (%)	Specificity (%)
		Lower Bound	Upper Bound			
HBD-3	0.93	0.85	0.99	< 0.0001	95	69
HBD-6	0.65	0.45	0.86	0.097	97	31
PCT	0.85	0.72	0.98	< 0.0001	97	53
CRP	0.78	0.64	0.92	0.0004	97	52

### Receiver operating characteristic analysis of protein concentration levels between cases and healthy controls

ROC analysis was performed to evaluate the diagnostic performance of HBD-3 and HBD-6 protein concentrations. Despite the limitations encountered in the quantification of PCT and CRP protein levels, ROC analysis of HBD protein concentrations was performed and the results are presented in **Table 6**. In patients with sepsis compared to healthy controls (**Table 6A**), only HBD-6 demonstrated a significant AUC (0.88; 95% CI: 0.76–0.99; *P* = 0.0004), indicating strong discriminatory capability. While HBD-6 exhibited high sensitivity (91%), its relatively low specificity (68%) highlights limitations in its diagnostic accuracy when used independently. A similar pattern was observed in patients with pneumonia (**Table 6B**), where HBD-6 again emerged as the only marker with a significant AUC (0.82; 95% CI: 0.66–0.98; *P* = 0.004), maintaining high sensitivity (91%) but demonstrating even lower specificity (42%).

**Table 6:** AUCs of HBD-3, HBD-6, PCT, and CRP protein for patients with sepsis, pneumonia, and pneumonia with sepsis, and healthy controls.

(A) protein (Sepsis)	AUC	95% CI		P values	Sensitivity (%)	Specificity (%)
		Lower bound	Upper bound			
HBD-3	0.61	0.44	0.79	0.214	94	36
HBD-6	0.88	0.76	0.99	0.0004	91	68
PCT	1	1	1	0.0017	75	100
CRP	1	1	1	0.083	67	100

(B) protein (Pneumonia)	AUC	95% CI		*P* Values	Sensitivity (%)	Specificity (%)
		Lower Bound	Upper Bound			
HBD-3	0.62	0.46	0.77	0.177	94	38
HBD-6	0.82	0.66	0.98	0.004	91	42
PCT	1	1	1	0.0012	75	100
CRP	1	1	1	0.0073	67	100

(C) Protein (Pneumonia with Sepsis)	AUC	95% CI		*P* Values	Sensitivity (%)	Specificity (%)
		Lower Bound	Upper Bound			
HBD-3	0.86	0.73	0.99	< 0.0001	94	48
HBD-6	0.89	0.78	0.99	0.0004	91	70
PCT	1	1	1	0.0016	75	100
CRP	1	1	1	0.049	67	100

Notably, in cases of co-infection (pneumonia with sepsis), all three HBDs showed significant AUC values (**Table 6C**). HBD-3 achieved the highest AUC (0.86; 95% CI: 0.73–0.99; *P* < 0.0001), along with excellent sensitivity (94%) and improved specificity (48%) compared to its performance in single-disease groups. HBD-6 also displayed robust diagnostic performance in co-infection scenarios (AUC = 0.89; 95% CI: 0.78–0.99; *P* = 0.0004), maintaining high sensitivity (91%) and demonstrating a promising specificity (70%).

Although the protein-level analysis of the established markers PCT and CRP could not be reliably assessed due to technical inconsistencies, the present findings highlight the diagnostic potential of HBD-6 in distinguishing both sepsis and pneumonia from healthy states. Additionally, HBD-3 emerges as a particularly promising candidate in the context of co-infection. HBD-6 showed the strongest diagnostic performance for sepsis and pneumonia, whereas HBD-3 demonstrated better performance in pneumonia-associated sepsis.

## Discussion

The main finding of this study was the significant upregulation of HBD-3 and HBD-6 mRNA expression in the peripheral blood of patients with sepsis, pneumonia, and pneumonia-associated sepsis compared with healthy controls, highlighting their potential as biomarkers of infection and sepsis. Increased expression of both defensins across all disease groups indicates activation of innate immune defense pathways in response to infection. Consistent with these findings, PCT and CRP mRNA expression were also significantly elevated in patients relative to healthy controls, with the highest levels observed in the pneumonia group. Collectively, these results suggest that HBD-3 and HBD-6 reflect systemic immune activation during infection and may serve as complementary biomarkers alongside established inflammatory markers, potentially improving the assessment of infectious and septic conditions. Human beta-defensins (HBDs) are small, cationic host defense molecules that play a vital role in the innate immune system. They exhibit broad-spectrum antimicrobial activity and contribute significantly to the body’s first line of defense against pathogens (28). HBDs are primarily expressed in epithelial cells at various mucosal surfaces, including the gastrointestinal tract, skin, respiratory tract, oral cavity, kidneys, nasal passages, eyes, mammary glands, and both the female and male genital tracts (29, 30). In the context of local infections, HBDs serve as crucial components of the biochemical barrier in epithelial tissues by interacting with microbial cell wall components, particularly membrane lipids, resulting in damage to microbial membranes. Beyond their antimicrobial function, HBDs are also involved in wound healing and exhibit anti-tumor properties (31). HBD-3 and HBD-6, members of the defensin gene family located on chromosome 8p23.1 (32), are natural antimicrobial peptides that additionally function as modulators of immune responses (33).

HBD-3 has broad antimicrobial activity against Gram-positive and Gram-negative bacteria and can modulate inflammation by interacting with lipopolysaccharide or limiting its interaction with host receptors (34, 35). In the lung, HBD-3 contributes to innate immune responses and is expressed in pulmonary epithelial cells and alveolar macrophages during infection with *Legionella pneumophila* infection (36). HBD-3 expression has also been reported in alveolar epithelial cells exposed to cigarette smoke extract (37) and in oral epithelial cells during HIV-1 infection, where it may interfere with viral replication through CXCR4-related mechanisms (38). Previous studies of HBD-3 in peripheral blood have shown variable findings, including results, with reports of undetectable expression in healthy individuals (39) and low expression in sepsis patients compared with controls (40). In contrast, the present study showed a significant upregulation of HBD-3 mRNA and protein levels in patient groups, particularly in pneumonia-associated sepsis, which. This discrepancy may reflect differences in patient populations, infection source, disease severity, sampling time, or assay methodology. Basal HBD-3 expression in non-inflamed tissues has also been suggested to contribute to immune homeostasis (41).

HBD-6 is less extensively studied than HBD-3 but has been implicated in innate immunity, inflammatory regulation, and reproductive tissue biology, with reported expression in the lung, epididymis, and testis (42–44). HBD-6 mRNA has been detected in the epididymis of a prostate cancer patient (45), and purified HBD-6 peptide shows antimicrobial activity against *Escherichia coli*, *Staphylococcus aureus*, and *Candida albicans* (23). Lower HBD-6 expression has also been reported in SARS-CoV-2-infected nasopharyngeal/oropharyngeal samples from individuals infected with SARS-CoV-2 (46). Given the limited data are available on HBD-6 in human infectious diseases, this study provides novel evidence that HBD-6 mRNA and circulating protein are increased in sepsis, pneumonia, and pneumonia-associated sepsis.

At the protein level, ELISA confirmed higher plasma concentrations of HBD-3 and HBD-6 in all patient groups compared with healthy controls (*P* ≤ 0.05), supporting the transcriptional activation of HBDs is reflected systemically at the circulating protein level. PCT and CRP were also elevated; however, technical limitations affected the reliability of protein-based interpretation of their protein-based diagnostic performance. This study relates to HBD-3 and HBD-6. Notably, HBD-6 protein showed stronger diagnostic performance than HBD-3 in sepsis and pneumonia, whereas both markers were informative in pneumonia-associated sepsis.

In response to immune activation, procalcitonin (PCT), a precursor of calcitonin, is synthesized by various tissues and organs during systemic infection (47). PCT mRNA is broadly expressed across multiple human tissues, with the highest levels detected in the liver, testis, lung, prostate, kidney, and small intestine (48). Human peripheral blood mononuclear cells (PBMCs) have been shown to express both PCT mRNA and protein, with expression modulated by lipopolysaccharide (LPS) and various sepsis-associated cytokines (49). Furthermore, PCT protein has been detected in the liver of individuals diagnosed with sepsis (50), as well as in patients who developed sepsis following thyroidectomy, where expression was notably elevated (51).

C-reactive protein (CRP) is an acute-phase inflammatory protein synthesized by hepatocytes, with its levels rising markedly in response to inflammation or infection (52). Elevated concentrations of both PCT and CRP have been previously reported in patients with sepsis and systemic inflammatory response syndrome (SIRS), compared to those without SIRS (53). Building on these findings, our study is the first to demonstrate the upregulation of PCT and CRP gene expression in sepsis, pneumonia, and pneumonia with sepsis patients relative to healthy controls.

The validation of reliable biomarkers for sepsis has been a major focus of recent research, with the goal of facilitating early intervention to reduce the high mortality rates associated with sepsis and pneumonia (54, 55). Among the most studied biomarkers, PCT has been shown to rise significantly during bacterial infections and is commonly used to guide antibiotic therapy decisions in patients with systemic infections (56). CRP, meanwhile, has been extensively associated with chronic inflammation, with elevated levels observed in patients with coronary heart disease and type 2 diabetes (57, 58). Additionally, increased CRP levels have been reported in patients with appendicitis, cholecystitis, pancreatitis, and meningitis (59). While both PCT and CRP are sensitive indicators of inflammation and infection, they lack the specificity needed to definitively distinguish sepsis from other conditions. To date, no single biomarker has demonstrated sufficient specificity for the early detection of sepsis or for differentiating it from related critical illnesses such as pneumonia (60–62).

Given their antimicrobial and immunomodulatory functions, HBDs may provide additional diagnostic information beyond conventional inflammatory markers. In this study, ROC curves were generated for both mRNA and protein levels to assess the diagnostic performance of HBD-3 and HBD-6 in comparison with PCT and CRP.

At the mRNA level, HBD-3 showed good discriminatory performance across all disease groups. In sepsis, HBD-3 achieved an AUC of 0.81, comparable to PCT and CRP, with high sensitivity but limited specificity. In pneumonia, HBD-3 also performed well (, an AUC = 0.88), while PCT and CRP demonstrated AUCs of 0.87 and 0.84, respectively. These results indicate that HBD-3 mRNA performs similarly to established biomarkers in distinguishing sepsis and pneumonia from healthy controls, although its moderate specificity limits its stand-alone diagnostic use.

The strongest mRNA-based performance was observed in pneumonia-associated sepsis, where. In this group HBD-3 achieved the highest AUC (0.93), with high sensitivity and improved specificity compared with its performance in sepsis or pneumonia alone. This suggests that HBD-3 mRNA may be particularly useful when where pneumonia and sepsis coexist. In contrast, HBD-6 mRNA showed lower AUC values across the studied conditions, despite high sensitivity, indicating that it may reflect infection-associated immune activation but is less suitable as a stand-alone mRNA diagnostic marker.

Protein-level ROC analysis showed a distinct different pattern. HBD-6 demonstrated significant diagnostic performance in sepsis and performance sepsis and pneumonia, with AUC values of 0.88 and 0.82, respectively. Although sensitivity was high, specificity remained moderate in sepsis and lower in pneumonia. In pneumonia-associated sepsis, both HBD-3 and HBD-6 proteins showed significant diagnostic value, with HBD-6 achieving the highest AUC (0.89) and better specificity than HBD-3. These findings suggest that HBD-6 protein may be more informative for distinguishing sepsis and pneumonia from healthy controls, whereas HBD-3 mRNA may be more useful in pneumonia-associated sepsis.

Taken together, these findings indicate that HBD-3 and HBD-6 may provide complementary diagnostic information.:. HBD-3 mRNA appears most useful in pneumonia-associated sepsis, whereas HBD-6 protein shows stronger performance in sepsis and pneumonia. However, both markers showed high sensitivity with moderate or low specificity, indicating potential which may increase the risk of false-positive results. Therefore, these HBDs HBD-3 and HBD-6 should not be considered replacements for current diagnostic approaches but may serve as adjunctive biomarkers to be when interpreted alongside clinical assessment, microbiological findings, and established inflammatory markers.

Previous studies have support investigated the diagnostic accuracy of PCT and CRP in sepsis and pneumonia. Tan et al. reported that PCT had superior diagnostic value compared with CRP in sepsis (60, 63), whereas CRP has been suggested to perform better than PCT for pneumonia diagnosis (60). PCT has also been used to help differentiate Gram-negative from Gram-positive bloodstream infections (64), and Maigari et al. reported that its usefulness that serum procalcitonin is useful for identifying and monitoring bacterial sepsis (65). The present findings are consistent with these observations and the established diagnostic value of PCT and CRP, but further suggest that HBD-3 and HBD-6 may add complementary information value information, particularly when pneumonia and sepsis overlap.

Overall, this study provides novel evidence that HBD-3 and HBD-6 are elevated at both mRNA and protein levels in sepsis, pneumonia, and pneumonia-associated sepsis. The findings support their potential use as adjunctive biomarkers, but validation in larger and more diverse cohorts is required. Future studies should also assess longitudinal changes in HBD levels to determine whether these markers can distinguish disease onset, progression, treatment response, and recovery.

### Study limitations

This study has several limitations. The sample size was modest, particularly for the pneumonia-associated sepsis group, which may limit statistical power and generalizability. The healthy control group differed in age from the patient groups, and comorbidities such as diabetes mellitus, hypertension, and dyslipidemia, may have influenced inflammatory biomarker levels. In addition, the heterogeneous microbiological profile, reflecting real-world ICU infections, may have contributed to variability in HBD expression. Technical inconsistencies also limited the interpretation of PCT and CRP protein measurements, preventing a full comparison with HBD protein performance. Despite these limitations, the study identifies HBD-3 and HBD-6 as promising adjunctive biomarkers that warrant further validation.

## Conclusion

This study investigated the potential of human beta-defensins (HBDs), particularly HBD-3 and HBD-6, alongside traditional biomarkers PCT and CRP, as diagnostic tools for sepsis, pneumonia, and pneumonia with sepsis. The results showed significant overexpression of mRNA for HBD-3, HBD-6, PCT, and CRP in the peripheral blood of affected patients, supported by elevated protein levels. ROC curve analyses revealed that HBD-3 and HBD-6 exhibited high sensitivity but moderate to low specificity, potentially leading to false positives. HBD-6 emerged as a promising biomarker, especially for distinguishing sepsis and pneumonia with sepsis from healthy controls. However, the study’s limited sample size and technical challenges in comparing protein levels for PCT and CRP highlight the need for further research. To strengthen the reliability of HBDs as biomarkers, future studies should increase sample size and refine detection methods, improving diagnostic accuracy for these critical conditions.

## Data Availability

The original contributions presented in the study are included in the article. Further inquiries can be directed to the corresponding authors.
