# Correction: Human beta defensins 3 and 6 serve as diagnostic tools for identifying sepsis and pneumonia

**DOI:** 10.3389/fimmu.2026.2011468

**Published:** 2026-10-02

**Authors:** Nourah Almansour, Fatema Al-Rashed, Shihab Kochumon, Ali Al Mahmeed, Eman Farid, Khalid Bindayna, Hasan Mohamed Saeed, Rasheed Ahmad

**Affiliations:** 1 Immunology and Microbiology Department, Dasman Diabetes Institute, Kuwait City, Kuwait; 2 Department of Microbiology, Immunology and Infectious Diseases, College of Medicine and Medical Sciences, Arabian Gulf University, Manama, Bahrain; 3 Adult Intensive Care Unit, Salmaniya Hospital, Ministry of Health, Manama, Bahrain

**Keywords:** biomarkers, diagnosis, HBD, pneumonia, sepsis

In the published article, the project number “RA-AM-2023–021” was incorrectly identified as a “Grant”. The correct designation is “Project No. RA-AM-2023-021”.

The correct **Funding** statement reads:

This study was mainly funded by the College of Medicine and Medical Sciences at Arabian Gulf University (Grant No. E021-PI-10/17), as well as by supporting from the Kuwait Foundation for the Advancement of Sciences (KFAS) and Dasman Diabetes Institute, Kuwait (Project No. RA-AM-2023-021).

The original version of this article has been updated.

